# Pulmonary Cryptococcosis in a Human Immunodeficiency Virus Negative Patient: A Case Report

**DOI:** 10.7759/cureus.9006

**Published:** 2020-07-05

**Authors:** Aakriti Soni, Pal Satyajit Singh Athwal, Sukhmanii Kahlon, Atul Gogia

**Affiliations:** 1 Internal Medicine, Himalayan Institute of Medical Sciences, Gurgaon, IND; 2 Internal Medicine, Saraswathi Institute of Medical Sciences, Hapur, IND; 3 Internal Medicine, Medical University of the Americas, Camps, KNA; 4 Internal Medicine, Sir Ganga Ram Hospital, New Delhi, IND

**Keywords:** pulmonary cryptococcal infection, hiv negative

## Abstract

Cryptococcosis is a major life-threatening fungal infection in patients with severe HIV infection and other immunocompromised states. Lung and central nervous system (CNS) are the most commonly involved organs in disseminated cryptococcosis. Others include skin, prostate, medullary cavity of bones, eyes, heart, liver, etc. Pulmonary cryptococcosis may be misdiagnosed because of comparatively nonspecific clinical and radiological features. We report the case of a 61-year-old male patient who is a known case of gastroesophageal reflux disease (GERD), myasthenia gravis, and steroid-induced diabetes mellitus. He was diagnosed with gangrenous cholecystitis at another institution but refused surgery. At our hospital, he experienced loss of consciousness in the out-patient department (OPD) and was therefore admitted for further evaluation where he was found to have pulmonary cryptococcosis and pancytopenia. Pulmonary cryptococcosis is usually found in HIV-positive immunosuppressed patients. However, sometimes it is also seen in HIV-negative patients, and they tend to have a good prognosis with adequate treatment.

## Introduction

Pulmonary cryptococcosis is an acute or chronic lung infection caused by different species of the encapsulated yeast, *Cryptococcus*, most commonly *Cryptococcus neoformans* or in some cases *C. gattii*. It may occur in an individual with normal immunity but tends to follow a very regressive course, as they are often asymptomatic and seldom seek any medical care. It is much more common in HIV-positive individuals and patients who have undergone organ transplantation. However, it may also be found in HIV-negative patients with other underlying immunosuppressive diseases. In these patients it shows a very accelerated course and may also lead to early dissemination. They may present with constitutional symptoms of fatigue and malaise and soon develop cough with sputum, pleuritic pain, and hemoptysis leading to acute respiratory distress syndrome (ARDS). The treatment is relatively long and may last for several months to a year. The HIV-negative patients are usually treated with amphotericin B with or without flucytosine followed by oral fluconazole [1].

## Case presentation

**Figure 1:**
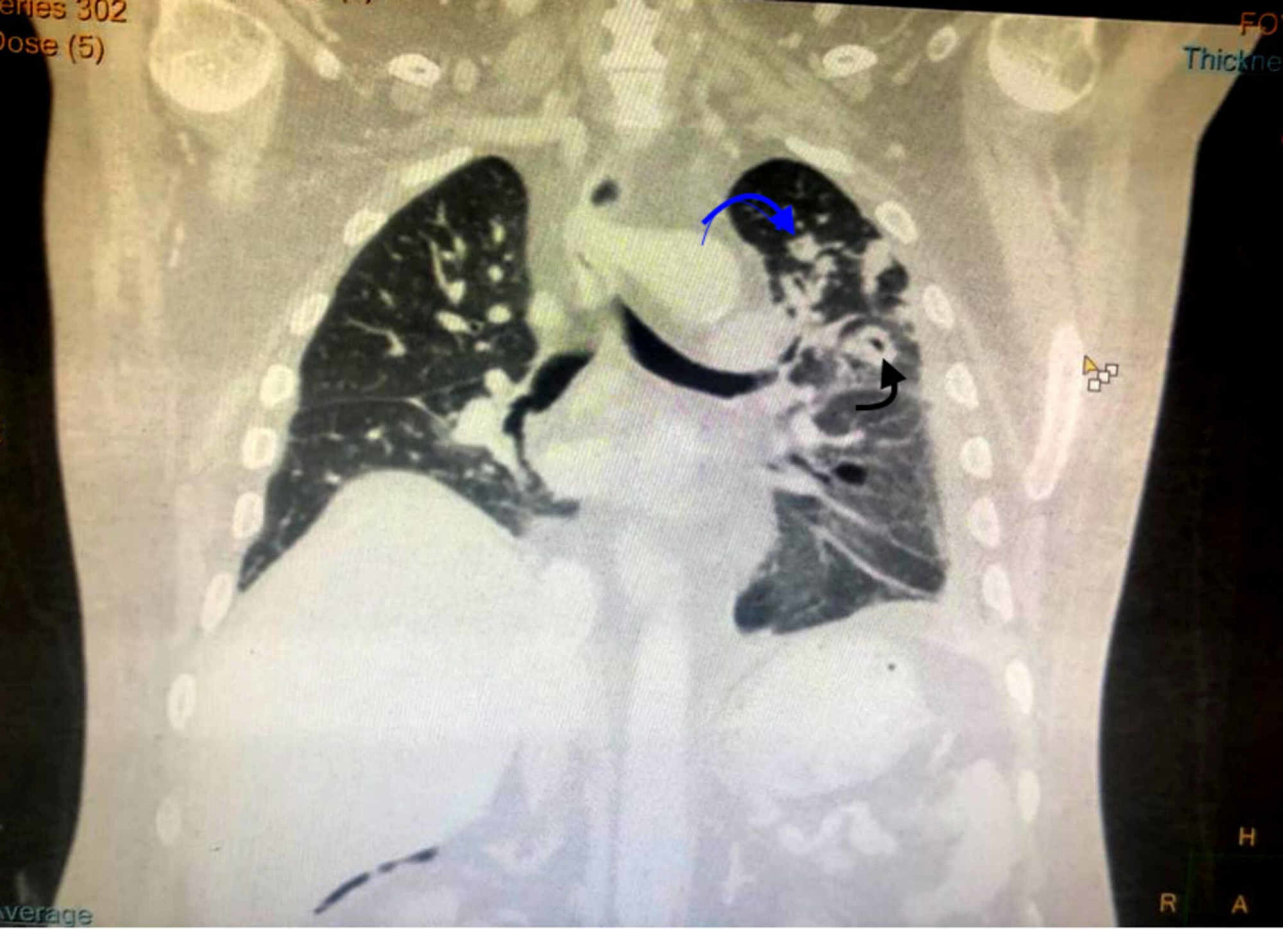
CT scan showing spiculated nodule demostrated by blue arrow and cavitations demonstrated by black arrow.

**Figure 2:**
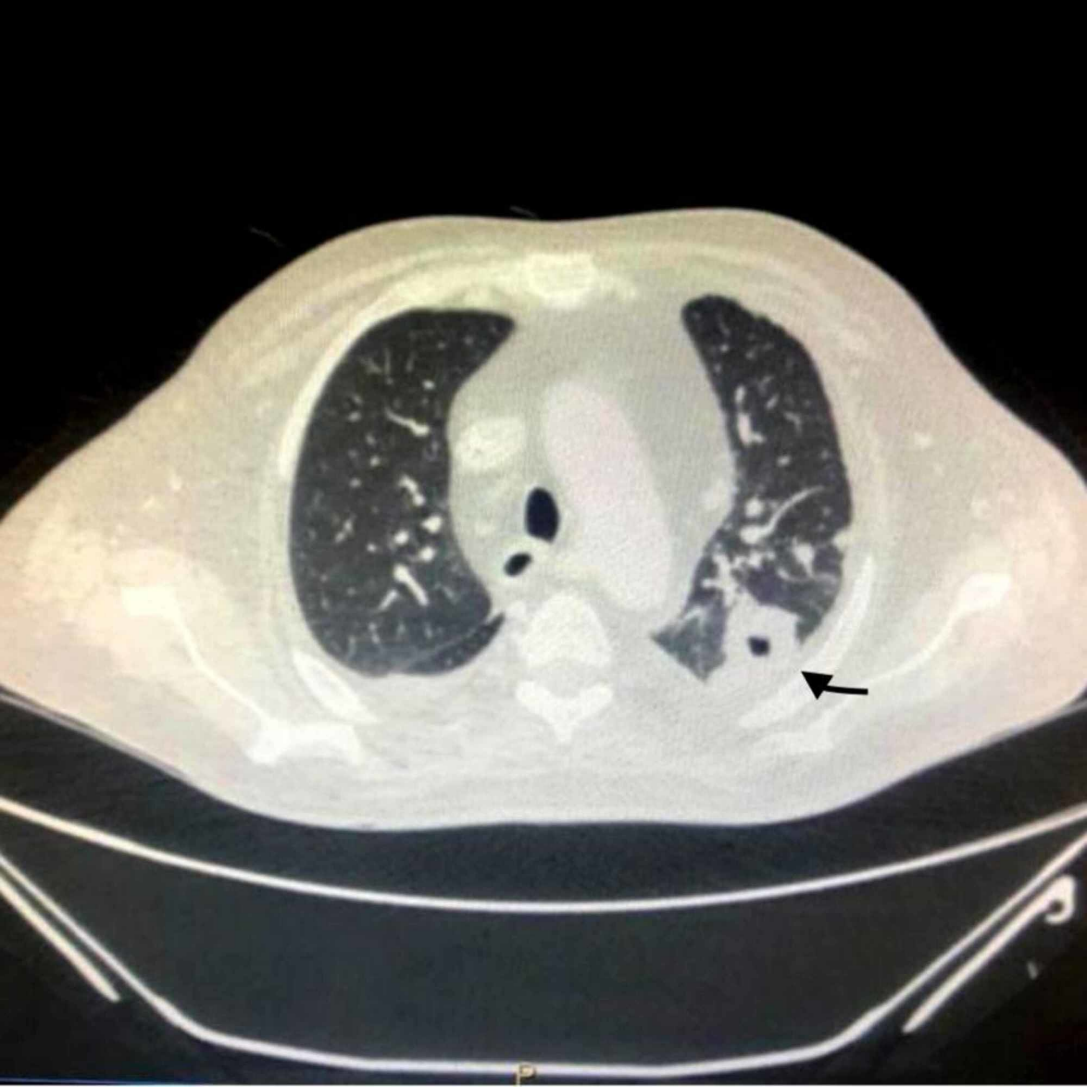
CT scan showing subpleural ground glass opacity and cavitation demonstated by black arrow.

A 61-year-old man with a history of gastroesophageal reflux disease (GERD), myasthenia gravis, and steroid-induced diabetes mellitus presented to our out-patient department (OPD) with complaints of weakness and dry cough with scanty sputum. He also had a history of weight loss of 20 kg over the past six months. He was diagnosed with gangrenous cholecystitis at another institution but refused surgery because of absence of symptomatology. He suffered loss of consciousness for two minutes there, which was not associated with incontinence or tonic-clonic seizure. There was no neurological deficit post the event, but he was still asked to get admitted for further evaluation. However, he left against medical advice and came to Ganga Ram Hospital, with the above-mentioned complaints. He developed fever on the day of the admission and was started on IV fluids, broad spectrum antibiotics, and insulin. He was found to have azathioprine-induced pancytopenia, hence it was stopped, and myasthenia was managed on pyridostigmine and low dose steroids. Chest X-ray (CXR) showed various nodular lesions suggestive of tuberculosis (TB). Further evaluation revealed negative mantoux and GeneXpert tests. A CT scan revealed bilateral pleural effusion (left>right) and multiple nodular lesions in the lung (Figures 1-2).

.

There was also evidence of ground glassing and atelectasis in both the lungs along with mediastinal lymphadenopathy. Abdominal ultrasound confirmed the presence of cholelithiasis with cholecystitis and free fluid in the lower abdomen and pelvis. Cardiac evaluation was essentially normal. An ultrasound guided cholecystostomy was done after explaining all the risks and taking an informed consent. The pus sample was sent for Gram, Zeihl-Nelson (ZN), and KOH stain along with culture sensitivity. It showed a growth of *Escherichia coli* and the treatment was modified in accordance with the sensitivity. The patient was transfused one unit of packed red blood cells (PRBC) in view hemoglobin value of 7.7 g/dL (normal value = 13.8-17.2 g/dL). His HIV and HCV (hepatitis C virus) status were nonreactive. His cough worsened and hence a bronchoscopy was planned, which revealed infective bronchial pathology in the form of thick, frothy, purulent secretions which grew *Cryptococcus* on bronchoalveolar lavage (BAL), however, were negative for acid fast bacilli. India ink staining confirmed it as *C. neoformans* and the patient was started on IV liposomal amphotericin B (AmBisome - 150 mg in 500 mL of 5% dextrose over 4-6 hours which was gradually increased to 250 mg over several days). Lumbar puncture performed ruled out cryptococcal meningitis. The patient also received daily nebulizations with salbutamol and ipratropium to relieve associated bronchospasm.

He developed bilateral lower limb edema due to hypoalbuminemia, as a result of the chronic infection, and was given human albumin 20% for the same. Treatment with anti-fungal showed improving clinical condition which was also correlated with improving chest radiography. Repeat abdominal ultrasound, however, revealed gall bladder distension with an 11 mm calculus, echogenic sludge in the lumen, and the tip of percutaneous catheter drainage (PCD) in situ. He underwent a laparoscopic cholecystectomy after an informed consent. He showed good clinical recovery postsurgery, but developed a tender and tense abdomen with fever on the fifth postoperative day. The bilious drain fluid was sent for culture and came positive for *Klebsiella pneumoniae*. Antibiotics were changed based on the sensitivity pattern. Endoscopic retrograde cholangiopancreatography (ERCP) and stenting were planned to correct the biliary leak. The patient was transfused four units of platelets before the procedure in view of thrombocytopenia (34000/mL, normal level = 1,50,000-4,50,000/mL). The patient had no new complaints, his blood counts showed an increasing trend, the peribag is okay, and there are no leaks seen. The abdomen is no longer tender and the patient shows improved spirometry results. He was advised to repeat a BAL but he opted against it. The patient was discharged on oral fluconazole (400 mg daily) and was asked to follow up after a month.

## Discussion

Cryptococcosis is a very common opportunistic infection seen in AIDS patients. It is also known to occur in other immunosuppressed patients and sometimes even in people with normal immunity [2]. Though the lungs and the central nervous system (CNS) are the most commonly affected, it is known to be aggressive and disseminates to involve almost any organ in immuncompromised patients. The most severe being meningoencephalitis, producing symptoms like headache, nausea, vomiting, convulsions, or even paralysis and coma. Inhalation of the yeast is the most common route of infection, yet pulmonary cryptococcosis is highly under- or misdiagnosed. This in part is due to the vague clinical presentations like subacute or chronic cough with sputum which can evoke a broad differential diagnosis, which is further complicated by occasional negative respiratory cultures [3]. It is most commonly confused with TB and *Pneumocystis jiroveci* infection. Radiography often fails to help make a clear diagnosis. The solitary/multiple nodules are indistinguishable from that of TB or a malignant tumor. Even 18F-fluorodeoxyglucose positron emission tomography/CT (18F-FDG PET/CT) does not help with an explicit diagnosis [4]. It is usually confirmed by microscopy of the pathological evidence or by identifying the actual fungus. Tissue obtained from the lung and thoracoscopic biopsies usually show the presence of cryptococcal granulomas. BAL washing culture sensitivity tends to be better than the biopsy specimens, showing encapsulated yeasts, and periodic acid-Schiff-positive bodies, hence confirming cryptococcal infection.

 The management protocol for pulmonary cryptococcosis in a HIV negative immunocompromised patient also takes around 10-12 months. Some 13 months after treatment cessation is when the antigen titers for cryptococcus tend to become negative. Presence of underlying immunocompromising states and meningoencephalitis greatly affect the choice of anti-fungal treatment and its duration [5].The treatment regimen for HIV-negative patients has not been fully explicated. Nevertheless, it is imperative for every patient with pulmonary or extra-pulmonary cryptococcosis to undergo a lumbar puncture to rule out associated CNS infection. For the immunocompetent, oral fluconazole, 200-400 mg/day for 3-6 months is enough but may extend to 12 months depending upon the severity of symptoms. Those who cannot tolerate fluconazole, they can be given itraconazole as a suitable alternative and certain patients where azole therapy cannot be given, amphotericin B (0.4-0.7 mg/kg/day, till a maximum of 1000-2000 mg) is used [6].

 In patients with underlying diseases, amphotericin B (liposomal, 0.7-1 mg/kg/day) for two weeks, followed by oral fluconazole (400-800 mg/day) for 8-10 weeks, followed by an even lower dose (200 mg/day) for one year is recommended as an appropriate line of treatment. Those patients receiving steroid along with the anti-fungal are usually shifted to a lower dose of steroid to ensure improved outcome [6]. Amphotericin B has a very troublesome side effect of causing nephrotoxicity, including hypokalaemia, renal tubular acidosis, etc. In contrast to the guidelines for pulmonary cryptococcosis in a HIV-negative patient, our patient received amphotericin B for six weeks. This extension was primarily due to the chronicity of his infection, the accompanying gall bladder perforation, and pancytopenia. According to Yamakawa, if a patient presents with a complex of anaemia, thrombocytopenia and ground glass consolidation, then pulmonary cryptococcosis should be given careful consideration [7]. No flucytosine was given, and he was finally discharged on oral fluconazole for at least a year. He refused to get BAL washings tested to check for a negative culture. He has been advised follow up, with a chest X-ray after one month.

## Conclusions

Pulmonary cryptococcosis is usually found in patients diagnosed with HIV or other immunocompromised individuals. It is rarely seen in HIV-negative patients, and is associated with high mortality without appropriate treatment. Further research is needed to form clear treatment protocol for cryptococcal disease in a HIV-negative patient. The cases in non-HIV patients are usually misdiagnosed with other infections like TB. Pulmonary cryptococcosis should be considered as one of the differentials irrespective of HIV or immune status of the individual. 
